# Lysine Crotonylation: An Emerging Player in DNA Damage Response

**DOI:** 10.3390/biom12101428

**Published:** 2022-10-05

**Authors:** Yuqin Zhao, Shuailin Hao, Wenchi Wu, Youhang Li, Kaiping Hou, Yu Liu, Wei Cui, Xingzhi Xu, Hailong Wang

**Affiliations:** 1 Beijing Key Laboratory of DNA Damage Response, College of Life Sciences, Capital Normal University, Beijing 100048, China; 2 Guangdong Key Laboratory for Genome Stability & Disease Prevention and Carson International Cancer Center, Marshall Laboratory of Biomedical Engineering, China Shenzhen University School of Medicine, Shenzhen 518060, China

**Keywords:** lysine crotonylation, DNA damage response (DDR), DNA double-strand break (DSB), DNA replication stress response

## Abstract

The DNA damage response (DDR) system plays an important role in maintaining genome stability and preventing related diseases. The DDR network comprises many proteins and posttranslational modifications (PTMs) to proteins, which work in a coordinated manner to counteract various genotoxic stresses. Lysine crotonylation (Kcr) is a newly identified PTM occurring in both core histone and non-histone proteins in various organisms. This novel PTM is classified as a reversible acylation modification, which is regulated by a variety of acylases and deacylases and the intracellular crotonyl-CoA substrate concentration. Recent studies suggest that Kcr links cellular metabolism with gene regulation and is involved in numerous cellular processes. In this review, we summarize the regulatory mechanisms of Kcr and its functions in DDR, including its involvement in double-strand break (DSB)-induced transcriptional repression, DSB repair, and the DNA replication stress response.

## 1. Introduction

DNA in cells is under constant threat from various genotoxic agents derived from endogenous cellular metabolism and the environment. These agents attack DNA to cause DNA damage, leading to the accumulation of lesions in each cell. These DNA lesions must be removed to ensure that cells function properly because they interfere with crucial functions of DNA, including replication and transcription. Failure to properly repair these DNA lesions can affect the stability of the genome, which is associated with cancer, developmental defects, infertility, immune deficiency, neurodegenerative diseases, and premature aging [1,2,3]. Mammalian cells have evolved a complex and versatile signaling network called DNA damage response (DDR) to mitigate the toxic effects of DNA lesions [4,5]. DDR is a concerted process involving the detection of DNA lesions, cell cycle checkpoint activation, DDR factor recruitment, chromatin reorganization, and DNA processing and repair. Initially, different DNA lesions are recognized by specific proteins that trigger and coordinate the recruitment of other DDR factors to DNA damage sites and initiate DDR [5,6,7]. With the recruitment of DDR factors, cascades of posttranslational modifications (PTMs), including phosphorylation, ubiquitylation, sumoylation, acetylation, and poly-ADP-ribosylation, are induced, which are associated with the modulation of related cellular processes such as the cell cycle, replication, transcription, and DNA repair [8,9,10,11,12,13]. Previous reviews have discussed in detail the mechanism of DDR and the crucial functions of various PTMs in DDR [4,7,11,14,15,16,17,18,19,20,21]. This review focuses on the emerging roles of lysine crotonylation (Kcr) in the modulation of DDR.

## 2. Kcr Is an Evolutionary Conserved and Abundant PTM

The roles of reversible acetylation of the ɛ-amino group of histone and non-histone lysines in DDR have been studied extensively [22,23,24,25,26,27]. In recent years, with the help of high-sensitivity mass spectrometry (MS), some short-chain acylation modifications, which also occur to the ɛ-group of lysine, including propionylation, butyrylation, 2-hydroxyisobutyrylation, succinylation, malonylation, glutarylation, crotonylation, β-hydroxybutyrylation, and lactylation, have been discovered successively. These acylation modifications are distinct in hydrocarbon chain length, hydrophobicity and charge but are structurally similar to the well-studied acetylation of lysine. These modifications also act like acetylation to neutralize the positive charge of the lysine side chain or to provide a larger lysine side chain for protein recognition and binding, thereby affecting the properties and functions of modified proteins [28,29,30,31,32,33,34,35]. Currently, the functional significance of these newly discovered PTMs, especially in DDR, remains largely unknown.

Kcr was first discovered as a new PTM of histone about ten years ago using an MS-based proteomics approach by Tan et al. [34]. In this study, Kcr was identified as a new type of PTM, 28 Kcr sites on core histones were discovered, and it was demonstrated that histone Kcr marks either active promoters or potential enhancers in both human somatic and mouse male germ cells [34]. This study opened a new direction in the field of PTMs and has attracted significant attention. Subsequently, histone Kcr was found to be associated with many diseases such as acute renal injury, depression, HIV latency, and the process of cancer. Kcr is found in different species, such as mouse, *Saccharomyces cerevisiae*, *Caenorhabditis elegans*, *Drosophila melanogaster*, and plants. Non-histone proteins have also been found to contain Kcr modifications. Numerous reports have shown that Kcr is an evolutionarily conserved and abundant PTM [36,37,38,39,40,41,42,43,44,45,46,47,48,49].

## 3. Enzymes Responsible for Reversible Kcr Regulation

Histone acetylation is one of the most widely studied epigenetic modifications related to chromatin remodeling, gene transcriptional regulation, and other key biological processes [50,51,52,53,54,55]. Protein lysine acetylations are regulated by the concerted actions of lysine acetyltransferases (KATs) (Kac “writer”) and lysine deacetylases (KDACs) (Kac “eraser”) that function by adding and removing acetyl groups from lysine residues, respectively. Because histones were the first identified Kac substrates, KATs and KDACs are often referred to as histone acetyltransferases (HATs) and histone deacetylases (HDACs), respectively. HATs/HDACs catalyze reversible acetylation of histone and non-histone proteins via transferring the acetyl group from acetyl-CoA to the ɛ-group of lysine or removing the acetyl group from a modified lysine [56,57,58,59,60,61,62,63,64]. Currently, no crotonyl-specific “writer” or “eraser” has been identified. Enzymes that regulate lysine acetylation also appear to regulate lysine crotonylation, although the two acyl groups are not identical in size and structure. The well-characterized HAT CBP (CREB-binding protein) and P300 have been shown to possess histone crotonyltransferase (HCT) activity and are possibly major HCTs in mammalian cells [65,66,67]. In addition, GNAT (GCN5-related N-acetyltransferase) family PCAF (P300/CBP-associated factor), MYST (Moz, Ybf2, Sas2, and Tip60) family hMOF (human males absent on the first), and HBO1 (histone acetyltransferase binding to ORC) were also revealed to possess HCT activity [68,69]. In mammals, Zn^2+^-dependent HDACs (HDAC 1–11, grouped into classes I, II, and IV) and NAD^+^ dependent sirtuins (SIRT 1–7, class III HDAcs) are responsible for removing acetyl groups from lysine residues in histone and non-histone substrates [70,71]. It is now clear that lysine crotonylation is also as dynamic as lysine acetylation. Class I HDACs (HDAC1/2/3) and the sirtuin family of HDACs (SIRT1/2/3/6) harbor histone decrotonyltransferase (HDCR) or lysine decrotonyltransferase (KDCR) activity, and class I HDACs are probably the major HDCR in mammalian cells [72,73,74,75,76]. Noteworthily, Wong’s lab generated CBP/p300 mutants with HCT but impaired HAT activity and HDAC1/HDAC3 mutants with DHCT but impaired DHAT, which may help to specifically study the function of histone Kcr and the molecular mechanism of its regulation [67,72]. Further studies are needed to determine whether these mutants apply to all histone Kcr sites and whether they also apply to non-histone proteins.

## 4. Regulation of Kcr by the Cellular Concentrations of Crotonyl-CoA

In vitro, acetyl-CoA and crotonyl-CoA can promote Kac and Kcr, respectively, without support from enzymes [77,78,79]. In cells, acetyl-CoA and crotonyl-CoA are necessary substrates for HATs to catalyze Kac or HCTs to catalyze Kcr. Acetyl-CoA is the most abundant CoA species measured, and crotonyl-CoA is approximately 1000-fold less abundant than acetyl-CoA in cells [66]. The relatively low intracellular abundance of crotonyl-CoA makes it easier to change the concentration of crotonyl-CoA than that of acetyl-CoA in cells and thus more likely to affect intracellular Kcr levels experimentally. Consistent with this, supplementation with crotonate significantly increased the cellular crotonyl-CoA and Kcr levels [34]. In contrast, depletion of acyl-CoA synthetase short-chain family member 2 (ACSS2), which converts crotonate into crotonyl-CoA, reduces the cellular crotonyl-CoA and Kcr levels [66]. Moreover, crotonyl-CoA is an endogenous intermediate metabolite during fatty acid oxidation and lysine/tryptophan metabolism. The key enzymes in these metabolic pathways that control crotonyl-CoA production can also affect the crotonyl-CoA and Kcr levels in cells. Depletion of mitochondrial short-chain acyl-CoA dehydrogenase (ACADS) or peroxisomal acyl-CoA oxidase (ACOX3), two key enzymes that catalyze the conversion of butyryl-CoA to crotonyl-CoA during fatty acid oxidation, also decrease the Kcr level by specifically reducing the cellular crotonyl-CoA level [80]. In addition to these enzymes capable of catalyzing crotonyl-CoA production, intracellular crotonyl-CoA can also be negatively regulated by crotonyl-CoA hydratase. Liu et al. reported that the chromodomain Y-like (CDYL) protein acts as a crotonyl-CoA hydratase to negatively regulate Kcr by converting crotonyl-CoA to β-hydroxybutyryl-CoA. CDYL transgenic mice showed dysregulation of Kcr and related phenotypes [79].

## 5. Recognition of Kcr by Chromatin-Associated Proteins

Increasing evidence suggests that crotonylation of histones is functionally different from histone acetylation and may regulate various cellular processes [81]. The identification of candidate proteins or structural modules that can “read” Kcr and translate Kcr of histones into diverse functional outcomes in cells has attracted much attention in the field. Chromatin-associated proteins are often involved in epigenetic regulation by recognizing PTMs of histones, such as phosphorylation and acetylation of histones, and thus were first investigated as Kcr reader candidates. Currently, no protein or structural module that specifically recognizes Kcr has been identified. Classical Kac “reader” proteins that contain YEATS (Yaf9, ENL, AF9, Taf14, and Sas5) and double plant homeodomain finger (DPF) domains preferentially bind Kcr when compared with Kac [82,83,84].

The YEATS domain, named for its five founding domain-containing proteins (Yaf9, ENL, AF9, Taf14, and Sas5), is evolutionarily conserved from yeast to human [85]. Originally, Li et al. reported that the YEATS domain of human AF9 strongly binds histone H3 acetylation at K9 (H3K9ac) and constitutes a novel family of histone Kac readers. Li et al. solved the crystal structure of the human AF9 YEATS domain bound to H3K9ac and found that the human YEATS domain organizes a unique aromatic “sandwich” pocket using highly conserved residues for Kac readout. The reader pocket of YEATS is characteristic of an “end-open” feature, indicating that it may accommodate the longer and more rigid Kcr better than Kac. This observation was confirmed by subsequent studies showing that Kcr enhances its binding to different YEATS domains by 2–5-fold when compared with Kac [82,86]. Structural analysis showed that AF9 YEATS uses the same Kac-binding aromatic sandwich pocket for Kcr recognition and the extended side chain of Kcr fits perfectly into the “end-open” pocket. The planar crotonylamide group is sandwiched by two aromatic residues that enable “aromatic-π-aromatic” stacking, which is optimal for Kcr recognition. The aromatic-π-stacking mechanism for Kcr recognition is observed consistently in the crystal structure of AF9, YEATS2, and Taf14 in complex with Kcr, and these YEATS domain proteins represent the first class of selective Kcr readers [82].

Although the “aromatic-π-stacking” mechanism perfectly accounts for the high affinity of the YEATS domain toward Kcr, it does not seem to be the recognition mechanism between all “readers” and Kcr. Recently, the DPF domains of the MYST family member monocytic leukemic zinc-finger (MOZ) and DPF2 were also found to recognize Kcr selectively. Crystal structures of the DPF/MOZ domain in complex with H3K14cr revealed that the DPF domain generates an intimate hydrophobic pocket without the participation of aromatic sandwiching residues to house the Kcr [87]. Recently, Klein et al. revealed another case of Kcr recognition by a DPF domain. The DPD domain of monocytic leukemic zinc-finger related factor (MORF) was also shown to bind H3K14cr over H3K14ac preferentially. The structure of the DPF/MORF-H3K14cr complex is very similar to the previously reported structure of the DPF/MOZ-H3K14cr complex, indicating that DPF/MORF and DPF/MOZ recognize Kcr by a conserved mechanism [88]. The DPF domains in MOZ and MORF represent the second class of selective Kcr readers.

## 6. Histone Kcr Is a New Determinant of Double-Strand Break (DSB)-Induced Transcriptional Silencing

PTM, such as phosphorylation, ubiquitination, ADP-ribosylation, and acetylation, plays a central regulatory role in the DDR network. Recent research has shown that Kcr is a new addition to the family. The dynamic changes induced by DNA damage are important indicators to evaluate whether a PTM is involved in DDR. Enas R. Abu-Zhayia et al. found that the Kcr level following laser microirradiation-induced DNA damage experienced a transient decrease [89]. DNA damage caused by ultraviolet radiation, ionizing radiation, and etoposide (VP16) in U2OS cells led to significant downregulation of H3K9cr levels in cells. Treating cells with trichostatin A (HDAC-specific inhibitor) but not nicotinamide (SIRT-specific inhibitor) increased the basal level of H3K9cr significantly and suppressed the reduction in H3K9cr after DNA damage. The authors concluded that the level of histone Kcr is downregulated by DNA damage and the decrotonylase activity of HDACs fosters this process. These works revealed for the first time the potential function of Kcr in DDR [89].

What specific functions does Kcr have in the process of the DSB response? Recent studies have shown that exogenous DSBs in the vicinity of transcriptionally active genes induce transient local transcriptional silencing at the DNA breakage site [90,91,92,93]. In general, transient transcriptional silencing near DSBs is postulated to be essential for avoiding clashes between transcription and DSB repair machinery and suppressing the generation of mutated RNA molecules when DSBs occur in the gene body. DSB-induced transcriptional silencing is instigated by Ataxia telangiectasia mutated (ATM), DNA-dependent protein kinase (DNA-PK) and poly-(ADP-ribose) polymerase 1 (PARP1) after DSB [91,92,94]. The downstream factors foster DSB-induced transcription silencing through direct inhibition of RNA Pol II mediated by negative elongation factor (NELF) or histone code editing mediated by CDYL1 and HDACs [90,93,95,96]. The molecular mechanisms underlying CDYL1-mediated transcriptional silencing after DSB have become increasingly clear. CDYL1 can be recruited rapidly to DSB damage sites in a PARP1-dependent manner. At the DSB site, CDYL1 performs transcriptional repressor functions via two different mechanisms.

**Figure 1:**
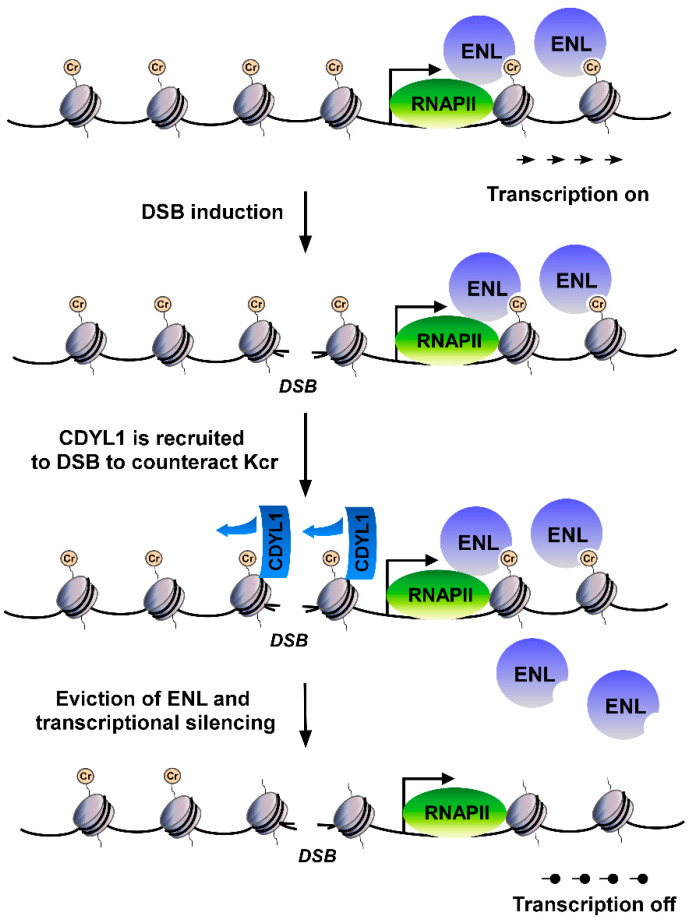
Functions of histone Kcr in DSB-induced transcriptional silencing. CDYL1 is rapidly recruited to the DSB sites when DSBs occur and counteracts the histone Kcr in the damaged region via its crotonyl-CoA hydratase activity. This activity leads to the eviction of the transcription elongation factor ENL and transcriptional silencing.

On the one hand, CDYL1 may achieve transcriptional repression by recruiting enhancers of zeste homolog 2 (EZH2) methyltransferase to DSB sites to promote local increases of the repressive methyl mark H3K27me3. On the other hand, CDYL1 can also downregulate Kcr at DSB sites via its crotonyl-CoA hydratase activity, resulting in the eviction of the transcription elongation factor ENL and transcriptional silencing [90,95,97]. Using ChIP-seq analysis, Enas R. Abu-Zhayia et al. found that the decrease in the levels of H3K9cr at DSB sites was accompanied by CDYL1 enrichment [97]. The decrease in Kcr levels in CDYL1-deficient cells is significantly lower than the decrease observed in CDYL1-proficient cells at DSBs. Crotonyl-CoA hydratase activity deficient mutant CDYL1-S467A was found to still promote DSB repair but lose the ability to promote Kcr downregulation and transcriptional repression at the DSB sites [97] (Figure 1). These results provide the first detailed explanation of the molecular mechanism of CDYL1 by which CDYL1-dependent Kcr participates in DSB-induced transcriptional silencing and suggest that homologous recombination-mediated repair and DSB-induced transcription silencing may occur independently. HDACs are also able to counteract Kcr after DNA damage. However, the mechanisms by which HDACs regulate Kcr at DSB and synergistically regulate DSB-induced transcriptional silencing with CDYL1 remain unresolved.

## 7. The Emerging Role of Kcr in DSB Repair

**Figure 2:**
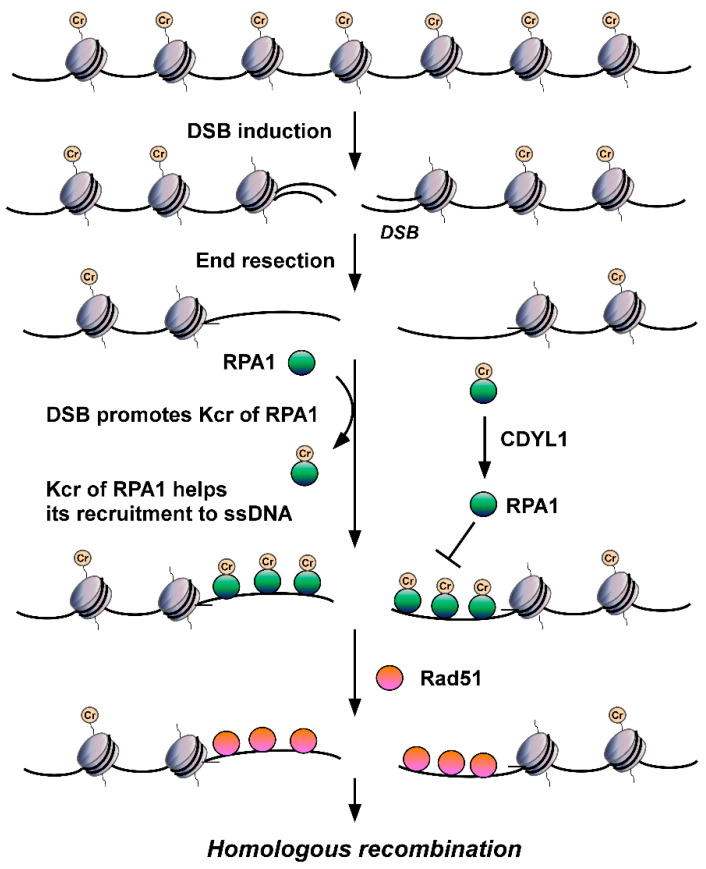
The roles of RPA Kcr in DSB repair. DSB injury promotes Kcr of RPA1. Kcr can increase the affinity of RPA for ssDNA, facilitate its recruitment to ssDNA generated by DSB end resection, enhance its interactions with major HR factors, such as RAD51, and promote HR-mediated DSB repair.

Is non-histone Kcr also involved in DDR? The answer is yes. The deletion of CDYL1 attenuates crotonyl-CoA hydrolysis, increases the global intracellular Kcr level, and then improves the detection of Kcr. Using quantitative MS to detect Kcr in CDYL1 deficient HeLa cells, Yu et al. identified a large crotonylome dataset containing 14,311 Kcr sites in 3734 proteins [98]. Using bioinformatics analysis, they found that Kcr levels of a series of protein factors related to DNA repair, including RPA1, POLD1, APEX1, XRCC5, and KDM1A, were increased significantly in CDYL1-deficient HeLa cells. They focused on RPA1 and found that CDYL1 negatively regulates K88cr, K379cr, and K595cr of RPA1. Further analysis showed that the Kcr level of RPA1 increases after hydroxyurea (HU), ultraviolet, ionizing radiation, VP16 or camptothecin insult, suggesting the level of RPA1 Kcr is upregulated by DNA damage and differs from the aforementioned histone Kcr. Through mutation analysis, Yu et al. hypothesized that Kcr of RPA1 enhances its interaction with single-strand DNA (ssDNA) (Figure 2) and major HR factors, such as MRE11 and BLM, and then promotes the recruitment of RAD51 recombinase and HR-mediated DSB repair [98]. These findings revealed for the first time the important roles of non-histone Kcr in DNA repair. According to Enas R. Abu-Zhayia et al., CDYL1 does not affect HR-mediated DSB repair through its crotonyl-CoA hydratase activity. In other words, CDYL1 does not regulate HR by influencing Kcr of RPA1 through modulating intracellular crotonly-CoA [97]. Other regulatory mechanisms, such as HCT/HDCR enzymes mediating crotonylation/decrotonylation, must be involved in DSBs damage-induced Kcr of RPA1 and subsequent regulated DSB repair, which need to be revealed in future efforts.

## 8. Kcr Is Involved in the Replication Stress Response

Slowed or stalled DNA replication forks may generate aberrant replication fork structures containing single-stranded DNA, which activate the replication stress response, a complex pathway mediated primarily by the kinase ATR (ATM- and Rad3-related). The correct response to replication stress can help cells to stabilize or restart stalled replication forks and avoid DNA damage and genomic instability. Our recent studies revealed that Kcr is involved in the replication stress response pathway in mammalian cells [74].

We focused on H2AK119cr (lysine crotonylation of H2A on lysine 119), a previously undefined histone Kcr. We found that H2AK119cr and H2AK119ub (mono-ubiquitination of H2A on lysine 119) exist simultaneously in cells and may influence each other. Replication stress, but not DSB damage, decreased the global levels of H2AK119cr and increased H2AK119ub in different cell lines. Reversible regulation of H2AK119cr and H2AK119ub under replication stress is governed by ATR but not ATM kinase. SIRT1 is responsible for the decrotonylation of H2AK119, whereas BMI1, a component of the canonical polycomb repressive complex 1 (PRC1), is necessary for the ubiquitination of H2AK119 during replication stress. Decrotonylation of H2AK119 by SIRT1 is a prerequisite for subsequent ubiquitination of this site by BMI1, and replication stress no longer promoted H2AK119ub in the absence of SIRT1 [74].

**Figure 3:**
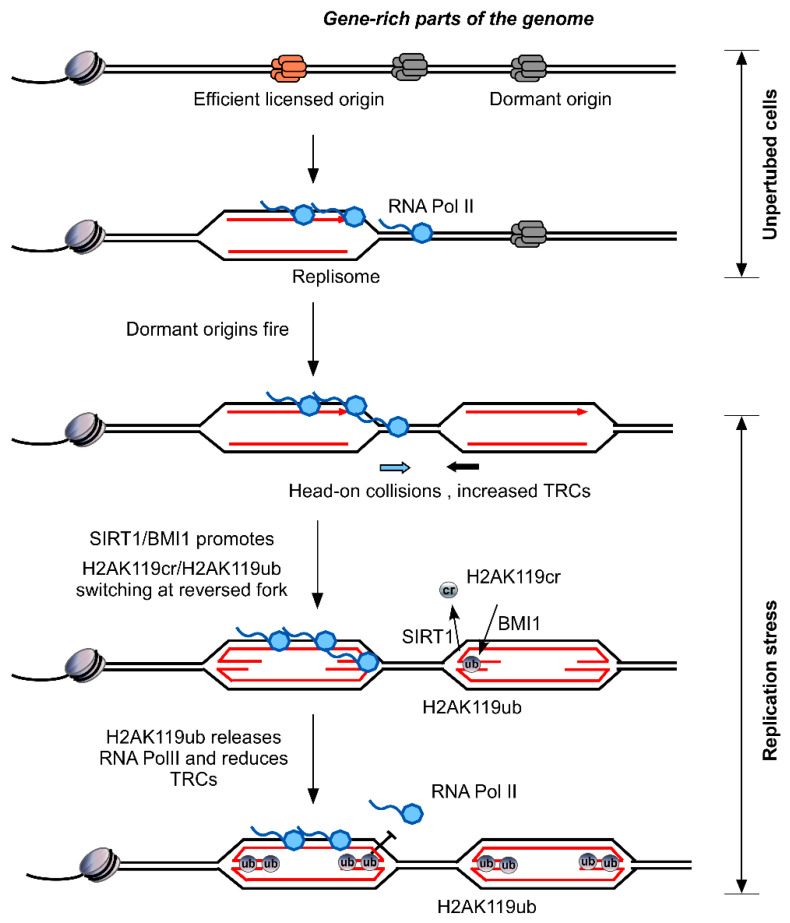
Dynamic switching of H2AK119cr/H2AK119ub reduces replication stress-induced TRCs. In unperturbed cells, active replication forks (derived from efficient active origins) passively replicate inactive origins (dormant origins) in the gene-rich part of the genome. Under replication stress, dormant origins are activated, increasing the chance that transcription will meet replication head-on, leading to more TRCs. Stalled forks undergo fork reversal. SIRT1/BMI1 mediate decrotonylation and ubiquitination of H2AK119 at reversed forks. Accumulating H2AK119ub at reversed forks leads to the release of RNA Pol II, resulting in transcriptional inhibition near the stalled replication fork and attenuation of TRCs.

Treating human cells with HU or doxorubicin may induce clusters of stalled replication forks in regions with actively expressed genes. The stalled replication forks undergo replication fork reversal, mediated by recombinase RAD51 and several translocases, to form a four-way Holliday junction-like structure by reconstructing the heterozygous region between the nascent DNA strands and the parent strands. Using Isolation of proteins on nascent DNA (iPOND) and an in situ proximity ligation assay (PLA), we demonstrated that H2AK119ub and H2AK119cr are enriched at reversed replication forks in wild-type and SIRT1 deficient cells, respectively. SIRT1/BMI1 regulates the dynamic switching of H2AK119cr/H2AK119ub at reversed forks upon replication stress (Figure 3).

RNA transcription and DNA replication are driven by different protein complexes, share the same DNA template, and in some cases they may conflict. Transcription replication conflicts (TRCs) can lead to the generation of excessive R-loops, affect the process of replication and transcription, induce stalling replication forks, and then cause genome instability. In human cells, transcription and replication normally proceed in the same direction, which allows the cell to minimize the TRCs and avoid the negative effects caused by the conflicts (Figure 3). Under replication stress, dormant origins near the stalled replication forks are activated to rescue stalled DNA replications. In regions of the genome containing actively expressed genes, the stalled replication forks and dormant origins fire increase the chance of head-on collisions between transcripts and DNA replicators, resulting in more TRCs and generation of the associated R-loops. Constant replication stress induces replication fork reversal, where SIRT1 and BMI1 are recruited to remove H2AK119cr and accumulate H2AK119ub in the nascent DNA. The enrichment of H2AK119ub in the active transcription region will prevent RNA Pol II from binding to the DNA template, resulting in RNA Pol II release, and thus attenuating the TRCs (Figure 3). These studies demonstrated that decrotonylation and ubiquitination of H2A at lysine 119 can help relieve replication stress induced TRCs, thereby protecting the stability of genome and revealing for the first time an important function of Kcr in the replication stress response. [74].

The roles of H2AK119cr on the reversed replication fork are still worth further investigation. It may be more than just an antagonistic mechanism of H2AK119ub involved in coordinating transcription and replication. In the absence of SIRT1, H2AK119cr accumulates at reversed forks and associates with newly synthesized DNA, suggesting that crotonylated H2A may be more suitable for nucleosome reassembly at the reversed forks. Whether and how histone Kcr participates in re-chromatinization at replication stress induced reversed forks is a fascinating question to be addressed. In addition, how cells recover from replicating stress responses is an interesting question. H2AK119cr may also accelerate cell recovery from replication stress response by reducing the inhibitory effect of H2AK119ub on actively transcribed genes. These studies will certainly expand Kcr-related research into more fields.

## 9. Conclusions and Perspectives

Increasing evidence shows that Kcr is an emerging player in DDR. Currently, we know that the level of Kcr is changed by DSB damage and replication stress and is involved in DSB repair, DSB-induced transcriptional repression, and resolving replication stress induced by TRCs. However, we believe these activities are just the tip of the iceberg, and many questions remain unknown and worthy of further exploration.

In recent years, high-sensitivity MS has helped to identify many Kcr sites, some of which are derived from DDR-associated proteins. However, because of the limitation of antibodies, the functional impact of these Kcr sites in DDR cannot be readily assessed. Only a few commercial antibodies that recognize specific sites are currently available, which greatly limits exploring the significance of Kcr in DDR. Therefore, an important future research direction is the development of more site-specific Kcr antibodies or new technologies to validate more Kcr sites sensitive to DNA damage and the different roles of these Kcrs in DDR.

Originally, Kcr was found to be mainly enriched in active promoters or potential enhancers to promote transcription, while our recent data indicate that H2AK119cr can also accumulate at reversed replication forks in the absence of SIRT1 [74]. This result suggests that H2AK119cr undergoes local enrichment at reversed replication forks. In the future, it will be interesting to explore whether other forms of chromosomal structural changes or chromosome remodeling induced by DNA damage or other reasons also lead to Kcr enrichment. Parsing their functions will expand our knowledge of Kcr and help us understand why some PTMs with low abundance also play important physiological functions.

Defects in the DDR system can lead to genetic disorders that predispose to cancer. Therefore, the mechanisms of Kcr action in DDR may also be used to develop novel cancer therapeutic strategies. Indeed, given that Kcr and Kac share some writers and erasers, it is possible that some anticancer drugs targeting Kac, such as HDACs inhibitors valproic acid and suberoylanilide hydroxamic acid, may derive part of their efficacy from affecting Kcr. Developing anti-cancer drugs specifically targeting Kcr or Kac is also a future research direction.
